# Effects of Pre-Competition Neuromuscular Electrical Stimulation Activation on Forward Lunge Performance and Neuromuscular Control in Squash Athletes: An Analysis Based on Timing and Electromyographic Sensors

**DOI:** 10.3390/s26123827

**Published:** 2026-06-16

**Authors:** Dongjin Li, Manxiu Bai, Haojie Li, Jian Jiang

**Affiliations:** School of Athletic Performance, Shanghai University of Sport, Shanghai 200438, China; lidongjin@sus.edu.cn (D.L.); matthew_manxiu_bai@163.com (M.B.); 202121070037@mail.bnu.edu.cn (H.L.)

**Keywords:** squash, neuromuscular electrical stimulation, forward lunge, electromyographic sensors, muscle synergy

## Abstract

Background: The Forward Lunge is a representative squash-specific footwork movement involving rapid acceleration, braking, postural stabilization, and return propulsion. This study examined whether pre-competition neuromuscular electrical stimulation (NMES) combined with weighted squats was associated with differences in Forward Lunge performance and neuromuscular control in squash athletes. Methods: Thirty-six male squash athletes were randomly assigned to three groups: Weighted Squats, Fake Stimulation, and Real Stimulation, with 12 participants in each group. After the assigned acute intervention, all participants completed the squash-specific star test. Completion time was recorded using a Microgate Witty photocell timing system, while surface electromyographic (sEMG) signals from 14 right-side muscles were collected using a Delsys Trigno wireless electromyography system. High-speed video was used to identify the Forward Lunge movement cycle, and transistor–transistor logic (TTL) synchronization enabled temporal alignment among timing, video, and sEMG signals. Normalized root mean square (RMS), muscle co-activation index (CI), and non-negative matrix factorization (NMF)-based muscle synergy parameters were calculated. Between-group differences were analyzed using one-way analysis of variance (ANOVA) with Bonferroni post hoc comparisons, and false discovery rate (FDR) correction was applied to secondary neuromuscular outcomes. Results: Star test completion time differed significantly among the three groups (F = 28.65, *p* < 0.001, η^2^ = 0.63). The Real Stimulation group showed a shorter completion time (10.35 ± 0.45 s) than the Weighted Squats group (11.80 ± 0.55 s) and Fake Stimulation group (11.55 ± 0.50 s). During the Forward Lunge movement cycle, normalized RMS values of the rectus abdominis (ABS; F = 18.56, *p* < 0.001, η^2^ = 0.55) and latissimus dorsi (LD; F = 13.42, *p* < 0.001, η^2^ = 0.44) were significantly higher in the Real Stimulation group. The gluteus maximus–biceps femoris (GLM–BF) co-activation index also differed significantly among groups (F = 58.42, *p* < 0.001, η^2^ = 0.78), with higher values in the Real Stimulation group. Muscle synergy analysis showed group differences in selected muscle activation weights and temporal activation parameters. Conclusions: In this parallel-group acute intervention study based on post-intervention measurements, real NMES combined with weighted squats was associated with shorter star test completion time and altered neuromuscular control during the Forward Lunge movement cycle. The integrated use of photocell timing, wireless sEMG, high-speed video, and TTL synchronization provided temporally aligned sensor-based evidence for evaluating acute pre-competition activation strategies. However, due to the absence of baseline measurements, the findings should be interpreted as post-intervention between-group differences rather than definitive evidence of individual improvement.

## 1. Introduction

Squash is a high-intensity intermittent racket sport characterized by repeated accelerations, decelerations, rapid changes in direction, and frequent lunging movements within a confined court space. During competition, players must respond rapidly to the opponent’s shot direction and move efficiently toward the front, lateral, and rear areas of the court while maintaining postural stability and technical readiness [1]. Among these movement patterns, the Forward Lunge is one of the most representative and frequently used footwork actions, particularly during front-court retrievals and rapid return movements [2]. The quality of the Forward Lunge depends on the athlete’s ability to accelerate toward the target, brake effectively during foot contact, stabilize the body during the support phase, and generate sufficient push-off force to return to the central position [3]. Although this movement appears to be dominated by lower-limb propulsion and braking, its successful execution requires coordinated activation of the lower limbs, trunk, and upper limbs. Therefore, examining the Forward Lunge from the perspective of both movement performance and multi-muscle coordination may provide a more comprehensive understanding of squash-specific neuromuscular control [4].

Pre-competition activation strategies are commonly used to acutely enhance neuromuscular readiness and subsequent sport-specific performance. Among these strategies, lower-limb loaded exercises, such as weighted squats, are widely used because they can increase motor unit recruitment, enhance muscle-tendon stiffness, and induce post-activation performance enhancement (PAPE) [5]. Previous studies have shown that appropriately prescribed resistance exercises may improve sprinting, jumping, change-of-direction ability, and explosive lower-limb performance by increasing neuromuscular excitability and contractile function [6]. However, the effectiveness of conventional weighted activation depends strongly on training status, load intensity, recovery interval, and the balance between potentiation and fatigue [7]. Excessive loading or insufficient recovery may impair subsequent movement performance because fatigue can offset the potential potentiation effect [8]. In squash, where athletes require rapid multidirectional movement and precise postural control, an activation strategy that enhances neuromuscular readiness without inducing excessive fatigue may be particularly important. Therefore, it remains necessary to explore whether combined activation methods can improve the acute effectiveness of conventional lower-limb weighted activation.

Neuromuscular electrical stimulation (NMES) has been increasingly used as an external neuromuscular activation method in sports training, rehabilitation, and performance enhancement contexts [9]. NMES can evoke involuntary muscle contractions through peripheral nerve stimulation, increase motor unit recruitment, and modulate neuromuscular excitability without relying solely on voluntary central drive [10]. Unlike voluntary resistance exercise, which generally follows a more orderly recruitment pattern driven by central motor commands, NMES may activate motor units through peripheral nerve pathways and provide an additional exogenous stimulus to the neuromuscular system [11]. Therefore, combining NMES with weighted squats may produce complementary acute effects: weighted squats may induce PAPE through voluntary high-force contraction and muscle-tendon potentiation, while NMES may further increase peripheral neuromuscular activation of targeted muscles. This combined stimulus may theoretically strengthen quadriceps activation and alter the trunk–lower-limb coordination demands required for braking, support stability, and push-off during the Forward Lunge. Previous studies have reported that NMES can influence muscle activation amplitude, force production, rate of force development, and neuromuscular coordination under certain stimulation parameters and task conditions [12]. However, whether pre-competition NMES combined with weighted squat activation can affect squash-specific star test performance and neuromuscular control during the Forward Lunge movement cycle remains unclear.

In addition, an appropriate control condition is necessary when evaluating NMES-based activation strategies. Sham stimulation can help control for non-specific effects associated with electrode placement, device operation, sensory input, and participant expectation. Therefore, comparing weighted squats alone, sham stimulation combined with weighted squats, and real NMES combined with weighted squats may help distinguish the effect of effective neuromuscular stimulation from the effects of loaded activation and stimulation-related expectancy.

With the development of wearable and laboratory-based sensing technologies, sensor-based assessment has become an important approach for quantifying sport-specific movement performance and neuromuscular function [13]. Timing sensors, such as photocell timing systems, can provide objective measurements of task completion time and are widely used to evaluate sprint, agility, and change-of-direction performance [14]. Surface electromyography sensors allow non-invasive recording of muscle activity during dynamic movement tasks and can be used to quantify muscle activation, co-activation, and multi-muscle coordination [15]. When combined with high-speed video and synchronization systems, timing and electromyographic sensors can provide temporally aligned information on external movement performance and internal neuromuscular activity within specific movement cycles [16]. This integrated sensor-based framework is particularly relevant for evaluating acute sport-specific activation strategies, because it links task-level performance outcomes with movement-cycle-specific neuromuscular control.

Beyond traditional muscle activation amplitude measures, muscle coordination analysis provides deeper insight into the organization of neuromuscular control during complex sport-specific movements. Muscle co-activation reflects the simultaneous activation of functionally related muscle pairs and is closely associated with joint stability, landing control, and movement stiffness regulation [17,18]. In addition, non-negative matrix factorization (NMF)-based muscle synergy analysis has been widely used to decompose multichannel electromyographic signals into low-dimensional coordination modules, thereby identifying muscle activation weights and temporal activation patterns [19]. Previous studies have suggested that changes in the number of muscle synergies, muscle weighting patterns, and activation timing may reflect task-specific motor control strategies and coordination demands [20]. During the Forward Lunge, effective movement performance requires coordinated activation of the hip, knee, ankle, trunk, and upper-limb muscles across the initiation, braking, support, and return phases. Therefore, combining RMS, co-activation, and muscle synergy analysis may provide a multidimensional representation of how NMES-based activation is associated with neuromuscular control in squash athletes.

Accordingly, the purpose of this study was to examine the effects of pre-competition NMES activation combined with weighted squats on Forward Lunge performance and neuromuscular control in male squash athletes using timing and electromyographic sensors. Specifically, this study compared three acute intervention conditions: weighted squats alone, sham stimulation combined with weighted squats, and real NMES combined with weighted squats. Star test completion time was used as the primary performance outcome, while surface electromyography-derived indices during the Forward Lunge movement cycle, including RMS, muscle co-activation, and muscle synergy characteristics, were used to evaluate neuromuscular control. It was hypothesized that real NMES combined with weighted squats would be associated with shorter squash-specific star test completion time and altered neuromuscular activation and coordination during the Forward Lunge movement cycle compared with weighted squats alone and sham stimulation. The findings may provide sensor-based evidence for optimizing pre-competition activation strategies in squash and offer practical implications for improving sport-specific movement performance through targeted neuromuscular stimulation.

## 2. Participants and Methods

### 2.1. Participants

An a priori sample size calculation was performed using GPower software 3.1. According to the experimental design of the present study, participants were randomly assigned to three experimental groups: Weighted Squats, Fake Stimulation, and Real Stimulation. The primary aim was to compare the effects of different acute interventions on star test performance during the Forward Lunge movement cycle in squash athletes. Therefore, the F tests family was selected in GPower, and ANOVA: Fixed effects, omnibus, one-way was used as the sample size estimation model. The parameters were set as follows: significance level α = 0.05, statistical power = 0.80, and number of groups = 3 [21]. The calculation indicated that a minimum total sample size of 30 participants was required. Considering potential participant dropout, invalid performance trials, or insufficient surface electromyography signal quality, 36 male squash athletes were ultimately recruited.

A total of 36 male squash athletes were included in this study. All participants had systematic squash-specific training experience and had achieved a top-three ranking in provincial or equivalent-level squash competitions, or possessed a comparable competitive level. After eligibility screening, participants were randomly allocated to the Weighted Squats, Fake Stimulation, and Real Stimulation groups, with 12 participants in each group. To better characterize the sample, demographic, anthropometric, training-related, and strength-related variables were recorded before the intervention, including age, height, body mass, body mass index, years of squash practice, weekly training volume, competitive level, relative squat strength, and previous resistance training experience. These variables were used to describe the baseline characteristics of the three groups and to evaluate the comparability of participants across groups. Participant characteristics are presented in Table 1.

The inclusion criteria were as follows: (1) male, aged 18–26 years; (2) systematic squash-specific training experience; (3) top-three ranking in provincial or equivalent-level squash competitions, or comparable competitive ability; (4) regular squash-specific training during the previous year; (5) no neurological, musculoskeletal, or cardiovascular disorders that could affect athletic performance or neuromuscular function; (6) no lower-limb sports injuries during the previous year that affected normal training or competition; (7) ability to perform weighted squats and the squash-specific star test in a stable and standardized manner; and (8) ability to understand the experimental procedures and complete all testing requirements.

The exclusion criteria were as follows: (1) failure to meet any of the inclusion criteria; (2) presence of neuromuscular disorders or chronic diseases that could affect motor control; (3) recent acute or chronic sports injuries, particularly involving the lower back, hip, knee, or ankle; (4) contraindications to neuromuscular electrical stimulation, such as a cardiac pacemaker, history of epilepsy, damaged skin at the stimulation site, or severe skin allergy; (5) high-intensity lower-limb resistance training, explosive training, or high-intensity squash competition within 48 h before testing; (6) inability to perform weighted squats or the star test according to the experimental requirements; and (7) voluntary withdrawal during the experiment or invalid key data due to equipment failure, electrode detachment, severe electromyographic noise, abnormal timing-gate recordings, or other technical issues.

Before the experiment, all participants were informed of the study purpose, experimental procedures, potential risks, and their rights. Written informed consent was obtained from all participants. The study was approved by the Ethics Committee of Shanghai University of Sport and was conducted in accordance with the ethical principles for human research.

### 2.2. Sensors and Testing Equipment

#### 2.2.1. Timing Sensors for the Star Test

**Figure 1:**
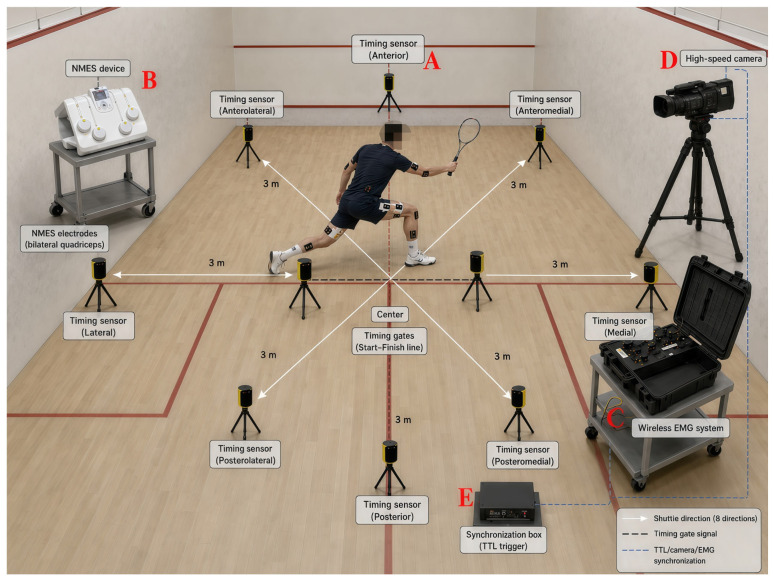
Schematic illustration of the testing equipment and experimental setup.

A Microgate Witty Timing System wireless photocell timing system (Microgate, Bolzano, Italy) was used to record the total completion time of the squash-specific star test. The system consists of wireless photocell sensors, reflectors, a timing unit, and data management software. During testing, the photocell sensors were positioned at the start/finish location of the star test and configured in start/stop trigger mode to automatically detect optical signal interruptions when participants crossed the start and finish points, thereby providing the total completion time of the star test (Figure 1A).

Participants started from the central testing area, completed the multidirectional movements along the prescribed star-shaped route in a fixed order, and returned to the start/finish area after completing all movement directions. The timing system recorded the elapsed time from the initial triggering of the start photocell to the final triggering of the finish photocell. In the present study, only the total completion time of the star test was extracted as the squash-specific performance outcome; split times were not analyzed. A shorter completion time indicated better squash-specific multidirectional movement speed, change-of-direction ability, and agility performance. Surface electromyographic signals and high-speed video data were collected synchronously during the star test. The Microgate Witty photocell sensors were used to quantify overall movement performance, the high-speed video system was used to identify the onset and offset events of the Forward Lunge movement cycle during the star test, and the surface electromyography sensors were used to record muscle activity of the target muscles within this movement cycle.

#### 2.2.2. Neuromuscular Electrical Stimulation Device

Neuromuscular electrical stimulation was delivered using a Compex SP 8.0 stimulator (Compex/DJO, USA). This device was used to administer the Fake Stimulation and Real Stimulation intervention conditions, whereas the Weighted Squats group did not receive electrical stimulation.

Self-adhesive electrodes measuring 5 cm × 5 cm were placed over the bilateral quadriceps region, mainly covering the muscle bellies and proximal motor point regions of the rectus femoris and vastus medialis. In the Real Stimulation group, effective neuromuscular electrical stimulation was applied with a stimulation frequency of 75 Hz, a pulse width of 400 μs, a duty cycle of 6 s:12 s, and an intensity set at 80–90% of each participant’s maximal tolerated intensity to elicit visible and tolerable muscle contractions. In the Fake Stimulation group, electrode placement, device connection, and experimental procedures were identical to those in the Real Stimulation group; however, stimulation intensity was maintained near or below the sensory threshold, producing only mild cutaneous sensation without visible muscle contraction (Figure 1B).

#### 2.2.3. Surface Electromyography Sensors

Surface electromyographic signals were collected using a Delsys Trigno Wireless EMG System (Delsys Inc., Natick, MA, USA). This wireless multichannel surface electromyography system enables synchronous recording of surface electromyographic signals from multiple muscles during dynamic movement tasks. In the present study, the sampling frequency was set at 2000 Hz, and the signal bandwidth was set at 20–450 Hz (Figure 1C).

#### 2.2.4. High-Speed Video System

Kinematic event identification during the Forward Lunge movement cycle was performed using a Photron FASTCAM Mini AX200 high-speed video system (Photron Limited, Tokyo, Japan). According to the requirements for identifying the onset and offset events of the Forward Lunge movement during the squash-specific star test, the frame rate was set at 200 Hz. The high-speed camera was positioned to fully capture the central testing area, the Forward Lunge movement path, and the return to the central area. It was used to continuously record the Forward Lunge movement during the star test. This system was not used to calculate the total completion time of the star test. Instead, it was used exclusively for offline frame-by-frame identification of key events within the Forward Lunge movement cycle, including movement onset, forefoot contact or support events, and cycle offset after the participant returned to the central area. Based on these identified time points, surface electromyographic signals within the corresponding movement cycle were extracted (Figure 1D).

#### 2.2.5. Multisensor Synchronization

To ensure temporal alignment among photocell timing data, surface electromyographic signals, and high-speed video data, a TTL trigger signal was used for multisystem synchronization. Before formal testing, the photocell timing system, surface electromyography system, and high-speed video system were connected through external trigger interfaces and configured with a unified trigger mode. At the start of each trial, the TTL signal simultaneously initiated performance timing, surface electromyography recording, and high-speed video recording, thereby establishing a common temporal reference point across all systems (Figure 1E).

### 2.3. Experimental Design and Stimulation Intervention Protocols

This study adopted a randomized controlled, parallel-group, acute intervention design. Participants were randomly assigned to the Weighted Squats, Fake Stimulation, or Real Stimulation group, with 12 participants in each group. All groups completed the same standardized testing procedures and multisensor data acquisition protocol, and no pre-intervention testing session was performed. Therefore, all between-group comparisons were based on post-intervention outcomes.

The experiment was conducted in a standard indoor squash facility using a squash-specific star test. Participants started from the center, moved to each target point in a prescribed order, touched or reached the target, and returned to the center until the full route was completed. The Forward Lunge was defined as the forward stepping movement from the center to the front-court target and back, and was selected for high-speed video event identification and surface electromyography analysis.

After screening, participants completed a standardized warm-up, squash-specific footwork familiarization, and MVC testing of the 14 target muscles. Following adequate rest, participants received the assigned acute intervention and immediately completed the star test within a standardized time window. Each participant performed six valid trials, with 60–90 s of rest between trials [22].

The Weighted Squats group performed three sets of five weighted squats at 60% 1RM, with 2 min of rest between sets, and received no electrical stimulation. This protocol was used as a conventional lower-limb weighted activation condition.

The Fake Stimulation group performed the same weighted squat protocol while receiving low-intensity sham stimulation. Electrode placement, device connection, and stimulation procedures were identical to those in the Real Stimulation group; however, stimulation intensity was maintained near or below the sensory threshold and did not induce visible muscle contraction. This condition was used to control for non-specific effects related to electrode attachment, device operation, participant expectation, and low-intensity sensory input.

The Real Stimulation group performed the same weighted squat protocol while receiving effective neuromuscular electrical stimulation. Stimulation was applied to the bilateral quadriceps, primarily targeting the rectus femoris and vastus medialis. The stimulation parameters were set as follows: frequency, 75 Hz; pulse width, 400 μs; duty cycle, 6 s:12 s; and intensity, 80–90% of each participant’s maximal tolerated intensity. The stimulation intensity was sufficient to elicit visible and tolerable muscle contractions. Electrical stimulation was delivered synchronously with the weighted squat task to provide external neuromuscular activation during the lower-limb loaded movement [23].

Thus, all three intervention protocols used weighted squats as the common movement basis and differed only in whether electrical stimulation was applied and whether the stimulation reached an effective neuromuscular activation intensity.

Participants were randomly assigned to the Weighted Squats, Fake Stimulation, or Real Stimulation group in a 1:1:1 ratio. The random allocation sequence was generated by an independent researcher who was not involved in participant recruitment, intervention delivery, data collection, or data analysis. Randomization was performed using a computer-generated random-number sequence. Group assignments were placed in sequentially numbered, opaque, sealed envelopes to ensure allocation concealment until the intervention session.

Because of the nature of the intervention, the researchers responsible for administering neuromuscular electrical stimulation could not be fully blinded to group allocation. However, participants in the Fake Stimulation and Real Stimulation groups received identical electrode placement and device procedures, and the Fake Stimulation protocol was designed to control for sensory input and expectation effects. The assessors responsible for high-speed video event identification and surface electromyography data processing were blinded to group allocation. All video and electromyography files were coded before analysis, and group labels were revealed only after data processing had been completed.

The sham stimulation protocol was designed to mimic the device setup and sensory experience of real neuromuscular electrical stimulation while avoiding effective muscle contraction. Participants in the Fake Stimulation group received the same electrode placement, device connection, body position, and intervention duration as those in the Real Stimulation group. Stimulation intensity was maintained near the sensory threshold to induce mild cutaneous sensation but no visible muscle contraction. This condition was used to control for non-specific effects related to electrode attachment, device operation, sensory input, and participant expectation. However, because visible muscle contraction was absent in the sham condition, complete participant blinding could not be fully guaranteed.

### 2.4. Selection of Target Muscles

The Forward Lunge is a representative front-court stepping movement in the squash-specific star test, consisting mainly of movement initiation, forward stepping, landing and braking, support stabilization, and push-off return. This movement requires not only the lower-limb muscles for propulsion, braking, and return push-off, but also the trunk muscles for postural stability, center-of-mass control, and force transmission, as well as the upper-limb muscles to assist balance and sport-specific movement posture. Therefore, the Forward Lunge can be used to characterize multi-muscle coordination during front-court movement and rapid return in squash athletes.

All participants in this study were right-side dominant. To minimize the influence of side-to-side differences on surface electromyographic signals and improve between-participant comparability, surface electromyographic signals were recorded from the right-side target muscles. Based on the movement characteristics of the Forward Lunge, squash-specific movement demands 14 right-side target muscles were selected: gluteus maximus (GLM), vastus medialis (VM), vastus lateralis (VL), biceps femoris (BF), medial gastrocnemius (GM), lateral gastrocnemius (GL), rectus abdominis (ABS), latissimus dorsi (LD), trapezius (TRAP), pectoralis major (PM), deltoid (DEL), biceps brachii (BB), triceps brachii (TB), and brachioradialis (BRD).

### 2.5. Segmentation of the Forward Lunge Movement Cycle

In this study, the Forward Lunge was defined as a complete movement in which participants performed a forward step from the central position toward the front-court target during the star test and then pushed off to return to the central area after touching or reaching the target. Following the concept of gait-cycle segmentation, one complete Forward Lunge movement cycle was defined as the period from the initiation of movement away from the central ready position to the return to the central area and re-establishment of a stable support posture [24].

The Forward Lunge movement cycle was identified offline frame by frame using high-speed video recordings. Movement onset was defined as the first frame in which the participant-initiated movement toward the front-court direction, indicated by initial lift-off of the stepping limb or a clear forward displacement of the center of mass. Movement offset was defined as the first frame in which the participant returned to the central area and re-established a stable double-leg support posture after completing the target-point task. Using the TTL trigger signal as the common temporal reference, the video-identified movement cycle was aligned with the surface electromyography time series. Surface electromyographic signals from the target muscles within this cycle were then extracted and time-normalized.

### 2.6. Data Processing and Analysis

#### 2.6.1. Performance Data Processing

The total completion time of the star test was automatically recorded and exported using the Microgate Witty Timing System. Before formal analysis, all test trials were screened for data quality. If a participant started prematurely, failed to complete the test according to the prescribed route, failed to touch the designated target point, lost balance during the test, or if photocell triggering failure or equipment-recording abnormalities occurred, the trial was considered invalid and was repeated after sufficient rest.

After completing the assigned acute intervention, each participant performed six valid star test trials. To reduce the influence of random error from a single trial, the mean completion time of the six valid trials was used as the final performance outcome for each participant. If any invalid trial occurred among the six trials, that trial was excluded and an additional trial was performed until six valid trials were obtained.

The total completion time of the star test was used as the primary performance outcome. A shorter completion time indicated better squash-specific multidirectional movement speed, change-of-direction ability, and agility performance.

#### 2.6.2. Surface Electromyography Signal Preprocessing

Surface electromyography signals were processed using MATLAB 2024b and Python 3.6. All raw electromyography data were checked for signal quality before analysis. Trials with obvious electrode detachment, signal saturation, severe motion artifacts, or abnormal synchronization signals were excluded from subsequent analysis. For each participant, six valid star test trials were retained after the assigned acute intervention, and one standardized Forward Lunge movement cycle was extracted from each valid trial for electromyographic analysis.

According to the onset and offset of the Forward Lunge movement cycle identified from high-speed video recordings, surface electromyographic signals from the 14 target muscles were extracted within the corresponding time window. The raw electromyographic signals were first demeaned to remove DC offset. A fourth-order zero-phase Butterworth band-pass filter was then applied with a frequency range of 20–400 Hz to reduce low-frequency motion artifacts and high-frequency noise. The filtered signals were full-wave rectified and further processed according to the specific analytical purpose for muscle activation, muscle co-activation, muscle synergy.

#### 2.6.3. MVC Normalization

To reduce the effects of between-participant differences in maximal strength, subcutaneous fat thickness, electrode placement, and muscle volume on electromyographic amplitude, maximum voluntary contraction (MVC) was used to normalize electromyographic amplitude. Before formal testing, participants completed MVC tests for the 14 target muscles. Each muscle was tested three times, with each trial lasting 3–5 s and a 30–60 s rest interval between trials [25].

The MVC signals were processed using the same preprocessing procedures as those used for the formal test signals, including demeaning, band-pass filtering, and full-wave rectification. For each muscle, the maximum electromyographic amplitude obtained from the three MVC trials was used as the MVC reference value. Electromyographic signals during the Forward Lunge movement cycle were divided by the corresponding MVC value and expressed as %MVC. The normalized electromyographic data were used for subsequent RMS, CI, and NMF analyses.

EMG_norm (t)=(EMG(t))/(EMG_MVC)×100%

where EMG_norm (t) represents the normalized electromyographic amplitude, EMG(t) represents the electromyographic amplitude at a given time point during the Forward Lunge movement cycle, and EMG_MVC represents the maximum electromyographic amplitude obtained from the MVC test for the corresponding muscle.

#### 2.6.4. Muscle Activation Analysis

The root mean square (RMS) amplitude was used to evaluate the activation level of each target muscle during the Forward Lunge movement cycle. RMS reflects the intensity of muscle activation and the effective amplitude of the electromyographic signal during the movement cycle. In this study, RMS was calculated for each target muscle during the Forward Lunge movement cycle based on the normalized electromyographic signal [26].

$$RMS=\surd (1/N\sum \_{\left(k=1\right)}^{N}=EMG\_norm\;{\left(k\right)}^{2})$$

where EMG_norm (k) represents the normalized electromyographic amplitude at the k-th sampling point, and N represents the number of sampling points within the Forward Lunge movement cycle. For each participant, RMS values were calculated for six valid Forward Lunge movement cycles, and the mean value across the six valid trials was used as the muscle activation outcome.

#### 2.6.5. Muscle Co-Activation Index Analysis

The co-activation index (*CI*) was used to evaluate the degree of simultaneous activation between functionally related muscle pairs during the Forward Lunge movement cycle. *CI* reflects the synchronized activation level between agonist–antagonist muscles or functionally synergistic muscles and can be used to analyze muscle coordination during support stabilization, landing braking, and push-off control in the squash-specific Forward Lunge movement [27].

*CI* was calculated based on the normalized electromyographic envelope. According to the movement characteristics of the Forward Lunge and the target muscles recorded in this study, the co-activation characteristics of the following functionally related muscle pairs were analyzed: VM–BF, VL–BF, GLM–BF, PM–LD, TB–BB, DEL–LD, and ABS–TRAP. VM–BF and VL–BF were used primarily to reflect co-activation between knee extensors and flexors during support and braking. GLM–BF was used primarily to reflect synergistic activation of hip extension-related muscles. PM–LD, TB–BB, DEL–LD, and ABS–TRAP were used primarily to reflect co-activation of upper-limb and trunk-related muscles during postural control, body balance, and force transmission.

$$CI=(2\int \_{\left(t\_1\right)}^{\left(t\_2\right)}=〖min[EM〗G\_1\;(t),EMG\_2\;(t)]dt)\;\;\;\;\;\;\;\;\;\;\;\;\;\;\;/(\int \_{\left(t\_1\right)}^{\left(t\_2\right)}=〖[EM〗G\_1\;(t)+EMG\_2\;(t)]dt)\times 100\%$$

where EMG_1 (t) and EMG_2 (t) represent the normalized electromyographic envelopes of the two target muscles during the Forward Lunge movement cycle, respectively, and t_1 and t_2 represent the onset and offset times of the movement cycle. For each participant, *CI* values were calculated for six valid Forward Lunge movement cycles, and the mean value was used as the muscle co-activation outcome.

#### 2.6.6. Non-Negative Matrix Factorization Muscle Synergy Analysis

Because the optimal number of extracted synergies may differ among participants and groups, the number of synergies and the overall variance accounted for were first compared among groups as independent synergy-related outcomes. For subsequent between-group comparisons of muscle weights and temporal activation parameters, only synergy modules that could be consistently identified and matched across all three groups were included in statistical analysis. Synergy modules that were not consistently present across all groups were described qualitatively and were not subjected to direct between-group statistical comparisons. This approach was adopted to avoid inappropriate comparison of non-equivalent or absent synergy modules across groups.

To determine the optimal number of synergy modules, the number of synergies was increased stepwise from 1 to 14, and NMF was performed separately for each value. After each decomposition, the variance accounted for (VAF) was calculated to evaluate the reconstruction quality. When the overall VAF first reached or exceeded 0.90, the corresponding minimum number of synergies was defined as the optimal number of synergy modules for that trial. If multiple numbers of synergy modules satisfied the VAF threshold, the smallest value reaching the threshold was selected to avoid over-decomposition.

The number of extracted synergies and overall VAF were used as the primary muscle synergy outcomes. Because the optimal number of synergies differed across participants and groups, direct statistical comparison of all extracted modules was not performed. Instead, synergy modules were first matched according to the similarity of muscle weight vectors, with additional consideration of the main contributing muscles and peak activation timing. Only modules that could be consistently identified and matched across all three groups were defined as common synergy modules and included in between-group statistical comparisons of muscle weights and temporal activation parameters.

Synergy modules that were not consistently present across all three groups were considered additional group-specific modules. These modules were described qualitatively in terms of their dominant muscle weights and activation timing but were not subjected to direct between-group statistical comparisons. This approach was adopted to avoid inappropriate comparison of non-equivalent or absent synergy modules across groups.

Non-negative matrix factorization (NMF) was used to analyze multi-muscle coordination during the Forward Lunge movement cycle. NMF decomposes multichannel surface electromyographic signals into a set of low-dimensional muscle synergy modules and allows extraction of the number of synergies, muscle synergy weights, and temporal activation parameters [28].

NMF analysis was performed based on the normalized electromyographic envelopes of the 14 target muscles. For each participant, six valid Forward Lunge movement cycles were retained under the assigned testing condition. Within each movement cycle, the electromyographic signals of each muscle were processed by demeaning, band-pass filtering, full-wave rectification, MVC normalization, smoothing, and time normalization, and were then arranged into a non-negative matrix V with dimensions of “muscles × time”. The rows of the matrix represented the 14 target muscles, and the columns represented the time-normalized sampling points. Matrix V was then decomposed into a muscle synergy weight matrix W and a synergy activation coefficient matrix H using NMF:

V_(m×n)=W_(m×k)H_(k×n)+E

where V is the normalized electromyographic envelope matrix, m is the number of muscles, n is the number of time-sampling points, k is the number of synergy modules, W is the muscle synergy weight matrix, H is the synergy activation coefficient matrix, and E is the reconstruction error matrix. Matrix W represents the relative contribution of each muscle to different synergy modules, whereas matrix H represents the temporal activation pattern of each synergy module during the Forward Lunge movement cycle.

To determine the optimal number of synergy modules, the number of synergies k was increased stepwise from 1 to 14, and NMF was performed separately for each k value. After each decomposition, the variance accounted for (VAF) was calculated to evaluate the degree to which the reconstructed matrix WH explained the original matrix V:

$$VAF=1-(\sum {\left(V-WH\right)}^{2})/(\sum V^{2})$$

When the overall VAF first reached or exceeded 0.90, the corresponding minimum k value was defined as the optimal number of synergy modules for that trial. If multiple numbers of synergy modules satisfied the VAF threshold, the smallest k value reaching the threshold was selected to avoid over-decomposition.

After determining the optimal number of synergy modules, the corresponding W and H matrices were extracted for subsequent analysis. To improve the comparability of muscle weights across trials and participants, the muscle weight vector of each synergy module was normalized as follows:

$$W\_(norm,j)=W\_j/\surd (\sum \_{\left(i=1\right)}^{m}=w\_ij^{2})$$

where W_j represents the muscle weight vector of the j-th synergy module, and w_ij represents the weight of the i-th muscle in the j-th synergy module. The normalized muscle weights were used to describe the relative contribution of each muscle to the corresponding synergy module.

The synergy activation coefficient matrix H was used to extract temporal activation parameters for each synergy module. The activation curve of each synergy module was expressed as a percentage of the Forward Lunge movement cycle. The peak activation time (T_max) was defined as the time point at which the activation coefficient of the synergy module reached its maximum value. The activation onset time (T_start) and activation offset time (T_stop) were determined using an activation threshold set at 20% of the peak activation amplitude of the corresponding synergy module. The first time point at which the activation coefficient exceeded this threshold was defined as T_start, and the last time point at which the activation coefficient remained above this threshold was defined as T_stop. Activation duration (T) was defined as the time interval between T_stop and T_start.

To ensure comparability of synergy modules across participants and trials, synergy modules were matched and ordered according to the similarity of their muscle weight vectors. Structural similarity between synergy modules was evaluated using Pearson correlation coefficients. Matching was performed primarily based on the similarity of muscle weight vectors; when the similarity of synergy structure was insufficient or multiple possible matches were present, the main contributing muscles and peak activation timing were also considered for module ordering.

The final muscle synergy outcomes used for statistical analysis included the optimal number of synergy modules, overall VAF, muscle weights of the matched synergy modules, peak activation time, activation onset time, activation offset time, and activation duration. For each participant, NMF analysis was performed on six valid Forward Lunge movement cycles under the assigned testing condition, and the mean value across the six valid trials was used as the participant’s muscle synergy outcome.

For muscle synergy extraction, the optimal number of modules was initially determined using a VAF-based criterion. The number of modules was increased stepwise, and the minimum number of modules at which the overall VAF exceeded 0.90 was identified. For the subsequent between-group comparison, SYN1–SYN4 were retained because the VAF exceeded 0.90 at four modules across all three groups. Therefore, the module number was variable during the exploratory extraction stage but fixed at four for the final comparison of equivalent synergy modules.

Synergy modules were aligned between participants and groups according to the similarity of muscle weight vectors. Pearson correlation coefficients were used as the primary criterion for module matching, with additional consideration of the dominant contributing muscles and peak activation timing. If a module could not be reliably matched to an equivalent module across groups, it was not interpreted as an independent group-specific statistical effect. This procedure was used to avoid inappropriate comparison of non-equivalent synergy structures.

### 2.7. Statistical Analysis

All statistical analyses were performed using SPSS 26.0. Continuous variables are presented as mean ± standard deviation. Before statistical analysis, data completeness, outliers, and distributional characteristics were examined. Normality was assessed using the Shapiro–Wilk test, and homogeneity of variance was assessed using Levene’s test. After the assigned acute intervention, each participant performed six valid star test trials, from which six valid Forward Lunge movement cycles were synchronously extracted. For each outcome, the mean value across the six valid trials was used as the final statistical value for each participant.

This study adopted a three-group randomized controlled, parallel-group design. The group factor included three levels: Weighted Squats, Fake Stimulation, and Real Stimulation. Because no pre-intervention testing session was included, all outcomes were compared among groups based on post-intervention data. Between-group differences were analyzed using one-way analysis of variance (one-way ANOVA). When a significant group effect was detected, Bonferroni-adjusted post hoc comparisons were performed. If the assumptions of normality or homogeneity of variance were not met, the Kruskal–Wallis test was used, followed by appropriate non-parametric post hoc comparisons.

Considering the multiple comparisons across RMS variables, muscle co-activation indices, muscle synergy weights, and synergy temporal parameters, the Benjamini–Hochberg false discovery rate (FDR) correction was applied separately within each outcome family. For muscle synergy analysis, the number of extracted synergies and overall VAF were first compared among groups. Because the optimal number of synergies could differ among participants and groups, only consistently matched common synergy modules were included in direct between-group statistical comparisons; additional modules were described qualitatively.

ANOVA results were reported as F values, *p* values, and η^2^. Pairwise comparisons were reported using adjusted *p* values, mean differences, and 95% confidence intervals where appropriate. For secondary neuromuscular outcomes, statistical significance was determined based on FDR-adjusted *p* values. The significance level was set at α = 0.05 [29]. For muscle synergy analysis, the number of extracted synergies and overall VAF were compared among groups. Because the optimal number of synergies differed across participants and groups, only consistently matched common synergy modules were included in direct statistical comparisons of muscle weights and temporal activation parameters. Additional group-specific modules were reported descriptively. FDR correction was applied to the statistical comparisons of synergy weights and temporal parameters.

## 3. Results

### 3.1. Star Test Performance

**Figure 2:**
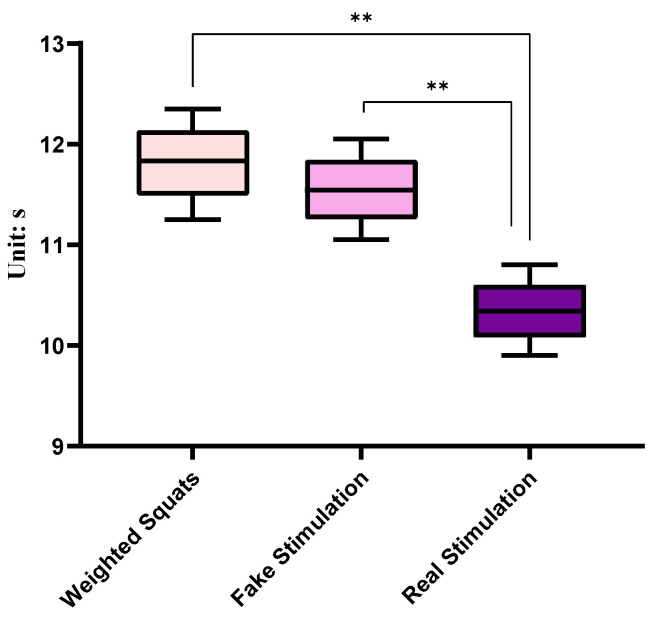
Results of star test performance. ** indicates a significant difference.

As shown in Table 2 and Figure 2, the total completion time of the squash-specific star test differed significantly among the three groups (F = 28.65, *p* < 0.001, η^2^ = 0.63). Post hoc comparisons showed that the Real Stimulation group completed the test in a significantly shorter time than both the Weighted Squats group and the Fake Stimulation group (*p* < 0.05). No significant difference was observed between the Weighted Squats and Fake Stimulation groups (*p* > 0.05).

### 3.2. Muscle Activation During the Forward Lunge Movement Cycle

**Figure 3:**
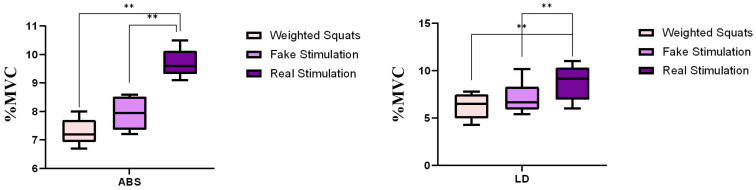
Results of RMS. ** indicates a significant difference.

As shown in Table 3 and Figure 3, RMS of the ABS differed significantly among the three groups during the Forward Lunge movement cycle (F = 18.56, *p* < 0.001, η^2^ = 0.55). Post hoc comparisons showed that RMS of the ABS was significantly higher in the Real Stimulation group than in the Weighted Squats and Fake Stimulation groups (*p* < 0.05), whereas no significant difference was observed between the Weighted Squats and Fake Stimulation groups (*p* > 0.05).

RMS of the LD also differed significantly among the three groups (F = 13.42, *p* < 0.001, η^2^ = 0.44). Post hoc comparisons showed that RMS of the LD was significantly higher in the Real Stimulation group than in the Weighted Squats and Fake Stimulation groups (*p* < 0.05), whereas no significant difference was observed between the Weighted Squats and Fake Stimulation groups (*p* > 0.05).

No significant between-group differences were observed in RMS for the remaining target muscles (*p* > 0.05).

### 3.3. Muscle Co-Activation During the Forward Lunge Movement Cycle

**Figure 4:**
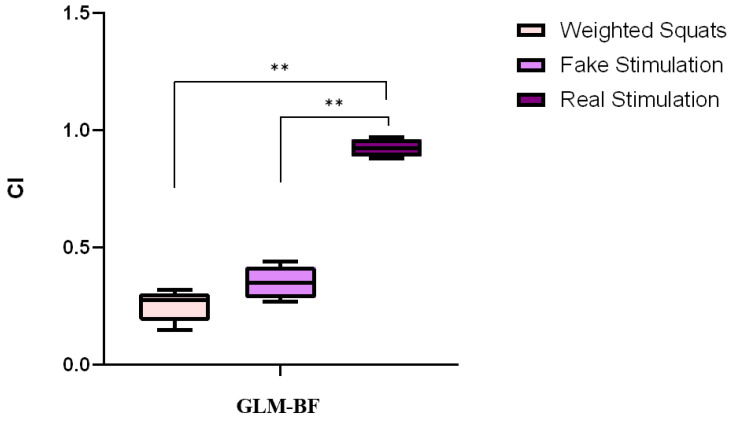
Results of CI. ** indicates a significant difference.

As shown in Table 4 and Figure 4, the co-activation index of GLM–BF differed significantly among the three groups during the Forward Lunge movement cycle (F = 58.42, *p* < 0.001, η^2^ = 0.78). Post hoc comparisons showed that the co-activation index of GLM–BF was significantly higher in the Real Stimulation group than in the Weighted Squats and Fake Stimulation groups (*p* < 0.05), whereas no significant difference was observed between the Weighted Squats and Fake Stimulation groups (*p* > 0.05).

No significant between-group differences were observed in the co-activation index for the remaining muscle pairs (*p* > 0.05).

### 3.4. Muscle Synergy Analysis During the Forward Lunge Movement Cycle

**Figure 5:**
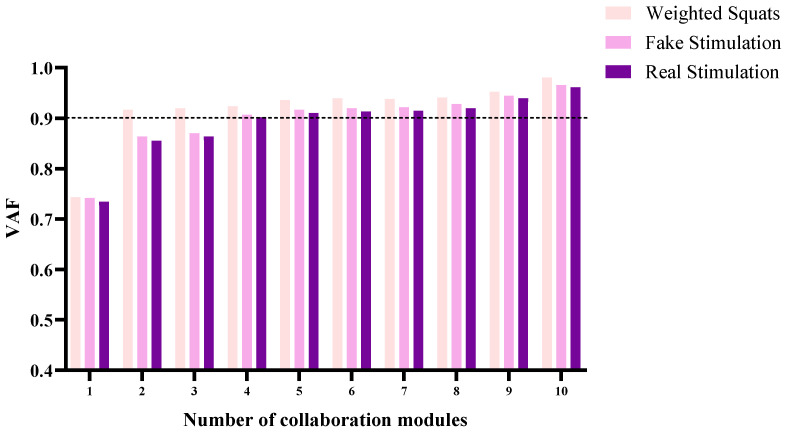
VAF values corresponding to different numbers of synergy modules.

For the muscle synergy analysis, the exploratory extraction showed that the number of modules required to reach the VAF threshold differed among participants and groups. However, the VAF exceeded 0.90 at four modules across all three groups; therefore, SYN1–SYN4 were retained for the between-group comparison of equivalent synergy modules. Modules were aligned according to muscle weight similarity, dominant muscle contribution, and peak activation timing. As shown in Table 5 and Figure 5, the NMF analysis showed that the numbers of muscle synergies extracted during the Forward Lunge movement cycle were 1.67 ± 0.82 in the Weighted Squats group, 4.83 ± 0.75 in the Fake Stimulation group, and 5.17 ± 0.41 in the Real Stimulation group. The changes in VAF corresponding to different numbers of synergy modules are presented in Figure 5.

**Figure 6:**
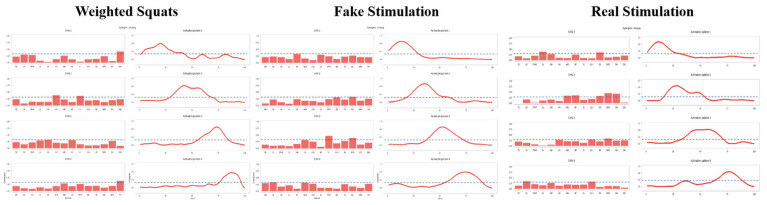
Muscle synergy weights and activation coefficient curves during the Forward Lunge movement cycle. The bar plots show the muscle weight distribution of each synergy module, and the line plots show the corresponding activation coefficient curves across the normalized Forward Lunge movement cycle. The horizontal dashed line indicates the activation threshold.

As shown in Table 6 and Figure 6, between-group differences were observed in the muscle weights of different synergy modules. In SYN1, the muscle weights of VL, GLM, and ABS were significantly higher in the Real Stimulation group than in the Weighted Squats and Fake Stimulation groups (*p* < 0.05). In SYN2, the muscle weights of ABS, LD, and DEL in the Real Stimulation group differed significantly from those in the Weighted Squats and Fake Stimulation groups (*p* < 0.05). In SYN3, the muscle weights of LD, TRAP, and BB were significantly higher in the Real Stimulation group than in the Weighted Squats and Fake Stimulation groups (*p* < 0.05). In SYN4, the muscle weight of BRD in the Real Stimulation group differed significantly from those in the Weighted Squats and Fake Stimulation groups (*p* < 0.05). No significant between-group differences were observed in the weights of the remaining muscles within each synergy module (*p* > 0.05).

**Table 6:** Comparison of muscle activation weights.

Muscle	Synergy	Weighted Squats	Fake Stimulation	Real Stimulation	Synergy	Weighted Squats	Fake Stimulation	Real Stimulation
GM	SYN 1	0.01 ± 0.04	0.15 ± 0.23	0.04 ± 0.04	SYN 2	0.00 ± 0.00	0.11 ± 0.13	0.08 ± 0.12
GL		0.01 ± 0.02	0.145 ± 0.26	0.05± 0.09		0.001 ± 0.002	0.08 ± 0.13	0.04 ± 0.04
VM		0.01 ± 0.01	0.11 ± 0.20	0.28 ± 0.35		0.00 ± 0.00	0.062 ± 0.247	0.204 ± 0.29
VL		0.00 ± 0.00	0.124 ± 0.333	0.205 ± 0.087 bc		0.017 ± 0.041	0.032 ± 0.268	0.213 ± 0.318
BF		0.056 ± 0.138	0.051 ± 0.090	0.202 ± 0.189		0.145 ± 0.355	0.051 ± 0.09	0.202 ± 0.189
GLM		0.106 ± 0.142	0.208 ± 0.246	0.359 ± 0.397 bc		0.073 ± 0.179	0.267 ± 0.22	0.175 ± 0.2
ABS		0.001 ± 0.001	0.036 ± 0.083	0.280± 0.163 bc		0.004 ± 0.011	0.128 ± 0.117	0.325 ± 0.432 bc
LD		0.27 ± 0.392	0.064 ± 0.086	0.121 ± 0.194		0.001 ± 0.002	0.116 ± 0.181	0.07 ± 0.075 bc
TRAP		0.054 ± 0.133	0.209 ± 0.216	0.201 ± 0.322		0.018 ± 0.044	0.211 ± 0.299	0.329 ± 0.391
BB		0.000 ± 0.00	0.193 ± 0.16	0.146 ± 0.222		0.004 ± 0.011	0.062 ± 0.313	0.091 ± 0.111
TB		0.043 ± 0.106	0.240 ± 0.262	0.052 ± 0.103		0.018 ± 0.045	0.077 ± 0.084	0.119 ± 0.141
DEL		0.00 ± 0.00	0.253 ± 0.249	0.094 ± 0.153		0.00 ± 0.00	0.106 ± 0.207	0.127 ± 0.156 bc
BRD		0.00 ± 0.00	0.204 ± 0.297	0.148 ± 0.279		0.021 ± 0.053	0.289 ± 0.315	0.170 ± 0.147
PM		0.059 ± 0.145	0.121 ± 0.166	0.016 ± 0.031		0.000 ± 0.00	0.060 ± 0.069	0.098 ± 0.204
GM	SYN 3	0.1510 ± 0.2371	0.1948 ± 0.2567	0.2271 ± 0.2731	SYN 4	0.0981 ± 0.2009	0.1603 ± 0.2734	0.256 ± 0.39
GL		0.239 ± 0.355	0.241 ± 0.297	0.255 ± 0.332		0.077 ± 0.117	0.140 ± 0.195	0.197 ± 0.241
VM		0.02 ± 0.045	0.099 ± 0.167	0.040± 0.097		0.175 ± 0.265	0.152 ± 0.264	0.093 ± 0.073
VL		0.00 ± 0.001	0.104 ± 0.195 a	0.042 ± 0.054		0.193 ± 0.284	0.136 ± 0.16	0.025 ± 0.021
BF		0.133 ± 0.196	0.302 ± 0.155	0.192 ± 0.14		0.024 ± 0.06	0.052 ± 0.092	0.225 ± 0.274
GLM		0.046 ± 0.112	0.105 ± 0.112	0.035 ± 0.03		0.005 ± 0.012	0.204 ± 0.235	0.211 ± 0.21
ABS		0.036 ± 0.088	0.122 ± 0.208	0.015 ± 0.024		0.025 ± 0.057	0.046 ± 0.075	0.109 ± 0.118
LD		0.00 ± 0.00	0.095 ± 0.146	0.221 ± 0.309 bc		0.005 ± 0.012	0.242 ± 0.383	0.202 ± 0.279
TRAP		0.02 ± 0.044	0.259 ± 0.392	0.32 ± 0.27 bc		0.00 ± 0.00	0.067 ± 0.104	0.026 ± 0.062
BB		0.018 ± 0.038	0.149 ± 0.087	0.242 ± 0.253 bc		0.039 ± 0.096	0.087 ± 0.085	0.176 ± 0.22
TB		0.030 ± 0.073	0.120 ± 0.205	0.137 ± 0.195		0.026 ± 0.063	0.174 ± 0.30	0.101 ± 0.227
DEL		0.020 ± 0.048	0.227 ± 0.232	0.177 ± 0.136		0.01 ± 0.202	0.024 ± 0.032	0.101 ± 0.09
BRD		0.042 ± 0.072	0.042 ± 0.065	0.251 ± 0.284		0.038 ± 0.054	0.052 ± 0.055	0.229 ± 0.153 bc

Note: Values are presented as mean ± standard deviation. Muscle activation weights indicate the normalized relative contribution of each muscle to each synergy module. Four synergy modules were retained for comparison because the VAF exceeded 0.90 at four modules across the three groups. Because of the large number of comparisons, significant post hoc differences are indicated by superscript letters, and *p*-values were adjusted using the Benjamini–Hochberg FDR procedure within the muscle synergy weight outcome family. a indicates Weighted Squats vs. Fake Stimulation; b indicates Weighted Squats vs. Real Stimulation; c indicates Fake Stimulation vs. Real Stimulation. GLM = gluteus maximus; VM = vastus medialis; VL = vastus lateralis; BF = biceps femoris; GM = medial gastrocnemius; GL = lateral gastrocnemius; ABS = rectus abdominis; LD = latissimus dorsi; TRAP = trapezius; PM = pectoralis major; DEL = deltoid; BB = biceps brachii; TB = triceps brachii; BRD = brachioradialis; VAF = variance accounted for. As shown in Table 7 and Figure 6, between-group differences were also observed in the temporal activation parameters of the synergy modules. In SYN1, activation duration T (F = 8.76, *p* < 0.001, η^2^ = 0.235), peak activation time Tmax (F = 31.24, *p* < 0.001, η^2^ = 0.523), and activation offset time Tstop (F = 31.72, *p* < 0.001, η^2^ = 0.527) were significantly lower in the Real Stimulation group than in the Weighted Squats and Fake Stimulation groups (*p* < 0.05), whereas no significant between-group difference was observed for Tstart (*p* > 0.05). In SYN2, activation duration T was significantly lower in the Real Stimulation group than in the Weighted Squats group (F = 25.10, *p* < 0.001, η^2^ = 0.468). Tstart (F = 14.10, *p* < 0.001, η^2^ = 0.331) and Tstop (F = 57.49, *p* < 0.001, η^2^ = 0.669) were significantly lower in the Real Stimulation group than in the Weighted Squats and Fake Stimulation groups (*p* < 0.05), whereas no significant between-group difference was observed for Tmax (*p* > 0.05). In SYN3, activation duration T was significantly higher in the Real Stimulation group than in the Weighted Squats and Fake Stimulation groups (F = 28.72, *p* < 0.001, η^2^ = 0.502). Tmax (F = 47.04, *p* < 0.001, η^2^ = 0.623) and Tstart (F = 150.64, *p* < 0.001, η^2^ = 0.841) were significantly lower in the Real Stimulation group than in the Weighted Squats and Fake Stimulation groups (*p* < 0.05), whereas no significant between-group difference was observed for Tstop (*p* > 0.05). In SYN4, activation duration T was significantly higher in the Real Stimulation group than in the Weighted Squats and Fake Stimulation groups (F = 66.49, *p* < 0.001, η^2^ = 0.700). Tmax (F = 62.66, *p* < 0.001, η^2^ = 0.687) and Tstart (F = 57.47, *p* < 0.001, η^2^ = 0.668) were significantly lower in the Real Stimulation group than in the Weighted Squats and Fake Stimulation groups (*p* < 0.05), whereas no significant between-group difference was observed for Tstop (*p* > 0.05).

**Table 7:** Comparison of activation coefficients.

Synergy	Parameters	Weighted Squats	Fake Stimulation	Real Stimulation	*p*-Value (FDR-Adjusted)	*p*-Value	η^2^
SYN1	Activation period T	35.67 ± 8.26	38.50 ± 5.54	26.50 ± 6.03 bc	8.76	<0.001	0.235
	Peak hours T_max_	23.52 ± 6.32	23.21 ± 4.12	13.30 ± 3.39 bc	31.24	<0.001	0.523
	Start time T_start_	0.10 ± 0.41	0.00 ± 0.00	0.52 ± 0.21	2.55	0.087	0.082
	End Time T_stop_	37.55 ± 3.14	39.17 ± 4.64	27.78 ± 4.48 bc	31.72	<0.001	0.527
SYN2	Activation period T	38.67 ± 4.32	30.67 ± 4.76	29.83 ± 3.82 b	25.10	<0.001	0.468
	Peak hours T_max_	36.52 ± 4.41	30.33 ± 7.78	31.21 ± 6.62	3.48	0.058	0.109
	Start time T_start_	27.79 ± 4.80	26.17 ± 4.71	21.18 ± 4.27 bc	14.10	<0.001	0.331
	End Time T_stop_	66.43 ± 5.21	52.21 ± 5.11	49.87 ± 5.58 bc	57.49	<0.001	0.669
SYN3	Activation period T	24.83 ± 5.57	30.33 ± 7.71	40.17 ± 4.71 bc	28.72	<0.001	0.502
	Peak hours T_max_	72.21 ± 3.38	72.52 ± 7.78	59.87 ± 5.90 bc	47.04	<0.001	0.623
	Start time T_start_	54.49 ± 2.22	47.78 ± 5.58	31.19 ± 4.42 bc	150.64	<0.001	0.841
	End Time T_stop_	76.91 ± 3.12	67.20 ± 5.55	71.12 ± 7.98	2.89	0.064	0.092
SYN4	Activation period T	19.83 ± 5.81	18.67 ± 4.63	28.67 ± 4.88 bc	66.49	<0.001	0.700
	Peak hours T_max_	86.79 ± 3.11	83.78 ± 5.21	72.29 ± 4.28 bc	62.66	<0.001	0.687
	Start time T_start_	74.11 ± 4.56	62.21 ± 7.70	60.09 ± 5.51 bc	57.47	<0.001	0.668
	End Time T_stop_	92.87 ± 3.21	93.33 ± 3.87	88.21 ± 4.48	2.13	0.128	0.070

Note: Values are presented as mean ± standard deviation. η^2^ = eta squared. *p*-values were adjusted using the Benjamini–Hochberg false discovery rate procedure within the RMS outcome family. b indicates a significant difference between the Weighted Squats and Real Stimulation groups; c indicates a significant difference between the Fake Stimulation and Real Stimulation groups after FDR correction.

## 4. Discussion

### 4.1. Effects of Real Stimulation on Star Test Performance

The present study showed that star test completion time differed significantly among the three groups. The Real Stimulation group completed the test faster than both the Weighted Squats and Fake Stimulation groups, whereas no significant difference was observed between the Weighted Squats and Fake Stimulation groups. This finding suggests that effective neuromuscular electrical stimulation combined with weighted squats may provide a greater acute enhancement of squash-specific movement performance than weighted activation alone or sham stimulation.

The star test involves repeated acceleration, braking, change in direction, and rapid return to the central position, which are key movement demands in squash. A shorter completion time therefore reflects better multidirectional movement speed and agility. The improved performance in the Real Stimulation group may be related to the additional neuromuscular activation induced by electrical stimulation during the squat task, which may enhance motor unit recruitment, peripheral excitability, and lower-limb readiness before subsequent high-speed movement [30].

This result is consistent with previous evidence indicating that appropriate pre-activation strategies can improve explosive and change-of-direction performance when potentiation exceeds fatigue [31]. However, weighted squats alone did not produce a clear advantage over sham stimulation in this study, suggesting that conventional loaded activation may be insufficient under some acute conditions. The addition of real stimulation may therefore strengthen the activation effect by providing an external neuromuscular stimulus, which is particularly relevant for squash movements requiring rapid propulsion, braking, and return during the Forward Lunge and star test.

### 4.2. Effects of Real Stimulation on Muscle Activation During the Forward Lunge

The present study found that the normalized RMS values of ABS and LD during the Forward Lunge movement cycle were significantly higher in the Real Stimulation group than in the Weighted Squats and Fake Stimulation groups, whereas no significant differences were observed in the remaining target muscles. This result suggests that real neuromuscular electrical stimulation combined with weighted squats was associated with selective changes in trunk-related muscle activation rather than a uniform increase in EMG amplitude across all recorded muscles.

During the Forward Lunge, ABS contributes to trunk stabilization and center-of-mass control, while LD is involved in trunk–upper limb force transmission and postural adjustment. Greater activation of these muscles may help athletes maintain body stability during forward stepping, landing braking, and push-off return. This may be particularly relevant in squash, where players must complete rapid lunging movements while maintaining balance and preparing for the subsequent shot.

This finding is partly consistent with previous evidence showing that acute neuromuscular activation strategies can influence task-specific muscle recruitment and postural control during dynamic movements [32]. However, the higher RMS values observed in ABS and LD should not be interpreted simply as evidence of greater neuromuscular efficiency. Increased muscle activation may reflect enhanced stabilization demands, but it may also indicate greater trunk stiffness, compensatory motor control, or task-specific adjustments used to maintain movement stability during the Forward Lunge. Although NMES was applied primarily to the quadriceps, the observed changes in ABS and LD suggest that real stimulation combined with weighted squats may have influenced whole-body neuromuscular organization through altered lower-limb activation and trunk stabilization requirements. Therefore, these results should be interpreted as evidence of altered neuromuscular control rather than definitive evidence of improved motor efficiency.

### 4.3. Effects of Real Stimulation on Muscle Co-Activation During the Forward Lunge

The present study found that the co-activation index of GLM–BF during the Forward Lunge movement cycle was significantly higher in the Real Stimulation group than in the Weighted Squats and Fake Stimulation groups, whereas no significant between-group differences were observed for the other muscle pairs. This result suggests that real neuromuscular electrical stimulation combined with weighted squats may selectively enhance hip-related muscle co-activation during the Forward Lunge, rather than increasing co-activation across all functional muscle pairs.

GLM and BF are both closely involved in hip extension, pelvic control, braking, and push-off during forward lunging movements. Increased GLM–BF co-activation may contribute to greater hip joint stability and more effective control of the center of mass during landing support and return propulsion. In squash, this may be particularly important because athletes must rapidly decelerate into a lunge and then recover quickly to the central position.

This finding is consistent with previous evidence suggesting that acute neuromuscular activation can enhance task-specific intermuscular coordination and joint stabilization during dynamic lower-limb movements [33]. Previous studies have also indicated that greater co-activation of functionally related muscles may improve movement stiffness regulation and support stability during high-speed change-of-direction tasks [34]. The absence of significant changes in other muscle pairs further indicates that the intervention effect was not a generalized increase in co-activation, but may reflect a targeted adjustment in hip-dominant neuromuscular coordination. This provides sensor-based evidence that real stimulation may modify specific coordination strategies during squash-specific Forward Lunge performance.

### 4.4. Effects of Real Stimulation on Muscle Synergy During the Forward Lunge

The present study found clear between-group differences in muscle synergy organization during the Forward Lunge movement cycle. The Real Stimulation group showed the highest number of extracted muscle synergies, followed by the Fake Stimulation group, whereas the Weighted Squats group showed the lowest number. In addition, Real Stimulation altered the muscle weighting patterns within several synergy modules, with greater contributions from VL, GLM, ABS, LD, TRAP, BB, DEL, and BRD in specific synergies. Significant differences were also observed in temporal activation parameters, indicating that real stimulation influenced not only the spatial composition of muscle synergies but also their activation timing.

A higher number of muscle synergies may reflect a more differentiated neuromuscular control strategy during complex sport-specific movement. The Forward Lunge requires rapid forward propulsion, landing control, trunk stabilization, upper-limb balance adjustment, and return push-off. Therefore, a more modular synergy structure may allow athletes to regulate different functional components of the movement more independently. Previous studies have shown that muscle synergy analysis can identify task-specific coordination modules and reveal changes in neuromuscular control that are not fully captured by single-muscle activation measures [35].

The increased contribution of lower-limb and trunk muscles in SYN1 and SYN2 may indicate enhanced involvement of propulsion, braking, and postural stabilization mechanisms. In contrast, the increased weights of LD, TRAP, BB, DEL, and BRD in later synergies may reflect greater upper-limb and trunk participation in balance regulation and movement reorganization during the return phase. This interpretation is consistent with evidence that complex dynamic movements require coordinated activation of lower-limb, trunk, and upper-limb muscles to maintain postural control and efficient force transmission [36].

The temporal activation results further suggest that Real Stimulation modified the timing strategy of coordinated muscle recruitment. Earlier activation timing in several synergy modules may support faster preparation for landing, braking, and return movement, whereas the longer activation duration in SYN3 and SYN4 may reflect sustained coordination demands during later phases of the Forward Lunge. Previous research has indicated that changes in synergy activation timing and duration can reflect adaptations in motor control strategy, movement complexity, and neural organization during dynamic tasks [37].

Compared with weighted squats alone, Real Stimulation may have provided additional peripheral neuromuscular input, thereby altering intermuscular coordination during the subsequent star test. Importantly, these changes were not limited to the stimulated quadriceps region but also involved trunk and upper-limb muscles, suggesting a broader whole-body coordination adjustment. The use of electromyographic sensor-based NMF analysis therefore provided a detailed view of how acute stimulation affects the spatial and temporal structure of neuromuscular control during squash-specific Forward Lunge performance [38].

Limitations: This study has several limitations. First, the parallel-group design included only post-intervention measurements; therefore, the results cannot confirm within-individual changes from baseline or fully establish causal effects of the intervention. Second, participant characteristics were relatively limited, and future studies should report more detailed information such as body mass, height, training experience, weekly training volume, competitive level, and strength background. Third, the sample included only male squash athletes, which may limit the generalizability of the findings. Finally, this study examined only acute responses; future studies with pre–post, crossover, or longitudinal designs are needed to verify the stability and practical value of this activation strategy. This study has several limitations. First, the parallel-group design included only post-intervention measurements; therefore, the results can demonstrate between-group differences after the intervention but cannot confirm individual changes from baseline or unequivocally establish intervention-induced improvement. Second, although additional demographic, anthropometric, training-related, and strength-related variables were reported to characterize the participants, no pre-intervention star test performance or electromyographic baseline was collected. Thus, potential baseline differences in sport-specific performance or neuromuscular patterns cannot be completely excluded. Third, although randomization, allocation concealment, and blinded video and electromyography analysis were applied, the researchers administering the stimulation could not be fully blinded because of the nature of the intervention. Finally, the sample included only male squash athletes, which may limit the generalizability of the findings to female athletes, youth athletes, or athletes from other competitive levels. In addition, the optimal number of extracted synergies differed across participants and groups. Therefore, only consistently matched common modules were included in direct statistical comparisons, whereas additional modules were described qualitatively. This approach reduced the risk of comparing non-equivalent modules but also limited the extent to which group-specific synergy structures could be statistically evaluated.

## 5. Conclusions

This study showed that, compared with weighted squats alone and sham stimulation, real neuromuscular electrical stimulation combined with weighted squats was associated with shorter squash-specific star test completion time, higher normalized RMS of selected trunk muscles, greater GLM–BF co-activation, and altered muscle synergy characteristics during the Forward Lunge movement cycle. Notably, the Real Stimulation group showed an approximately 12.3% shorter star test completion time than the Weighted Squats group, suggesting potential practical relevance for acute pre-competition activation in squash athletes. From a sensor-based assessment perspective, the integration of photocell timing sensors, high-speed video, wireless surface electromyography, and TTL synchronization enabled precise temporal alignment between external movement performance and EMG signals within the Forward Lunge movement cycle. This multi-sensor approach provided objective evidence for linking squash-specific performance outcomes with movement-cycle-specific neuromuscular control characteristics. However, because this study used a parallel-group design with post-intervention measurements only, the findings should be interpreted as between-group differences rather than definitive evidence of within-individual improvement caused by the intervention. In addition, this study did not include an NMES-only group, recorded EMG signals only from the right side, and applied stimulation only to the quadriceps. Future studies should adopt pre–post or crossover designs, include additional intervention arms, record bilateral EMG activity, and examine whether stimulation of other muscle groups produces different neuromuscular responses during squash-specific movements.

## Figures and Tables

**Table 1:** Participant characteristics.

Variable	Weighted Squats (*n* = 12)	Fake Stimulation (*n* = 12)	Real Stimulation (*n* = 12)
Age (years)	22.35 ± 1.56	21.89 ± 1.42	22.10 ± 1.71
Height (cm)	178.42 ± 5.63	177.85 ± 6.12	178.10 ± 5.78
Body mass (kg)	72.36 ± 6.84	71.92 ± 7.15	72.58 ± 6.47
Body mass index (kg/m^2^)	22.75 ± 1.68	22.71 ± 1.74	22.87 ± 1.61
Years of squash practice (years)	6.58 ± 1.31	6.42 ± 1.24	6.67 ± 1.37
Weekly training volume (h/week)	10.25 ± 1.86	10.08 ± 1.73	10.42 ± 1.62
Relative squat strength (1RM/body mass)	1.52 ± 0.18	1.49 ± 0.21	1.51 ± 0.19
Resistance training experience (years)	3.25 ± 0.87	3.08 ± 0.79	3.17 ± 0.83
Competitive level	Provincial top-three or equivalent	Provincial top-three or equivalent	Provincial top-three or equivalent

Values are presented as mean ± standard deviation or descriptive categories. 1RM = one-repetition maximum.

**Table 2:** Comparison of star test performance among the three groups.

Variable	Weighted Squats	Fake Stimulation	Real Stimulation	F-Value	*p*-Value (FDR-Adjusted)	η^2^
Total time (s)	11.80 ± 0.55	11.55 ± 0.50	10.35 ± 0.45 bc	28.65	<0.001	0.63

Values are presented as mean ± standard deviation. η^2^ = eta squared. *p*-values were adjusted using the Benjamini–Hochberg false discovery rate procedure within the RMS outcome family. b indicates a significant difference between the Weighted Squats and Real Stimulation groups; c indicates a significant difference between the Fake Stimulation and Real Stimulation groups after FDR correction.

**Table 3:** Comparison of RMS values of target muscles during the Forward Lunge movement cycle among the three groups.

Muscle	Weighted Squats	Fake Stimulation	Real Stimulation	F-Value	*p*-Value (FDR-Adjusted)	η^2^
GLM	8.0 ± 1.8	8.5 ± 1.9	9.8 ± 2.7	2.34	0.056	0.15
VL	7.8 ± 1.8	7.6 ± 1.9	8.2 ± 2.9	0.52	0.598	0.03
VM	7.5 ± 1.7	8.0 ± 1.8	8.5 ± 3.0	1.02	0.368	0.06
BF	6.8 ± 1.8	7.7 ± 1.9	8.0 ± 2.5	2.28	0.074	0.12
GM	7.2 ± 1.7	7.6 ± 1.8	7.9 ± 2.3	1.47	0.240	0.08
GL	6.9 ± 1.8	7.3 ± 1.9	7.6 ± 2.4	1.08	0.348	0.06
PM	7.8 ± 1.7	8.2 ± 1.9	9.5 ± 3.0	2.45	0.061	0.16
ABS	7.0 ± 1.6	7.5 ± 1.8	9.4 ± 3.1 bc	18.56	<0.001	0.55
LD	6.2 ± 1.4	7.0 ± 1.7	8.4 ± 2.6 bc	13.42	<0.001	0.44
TRAP	6.5 ± 1.8	6.0 ± 1.9	5.3 ± 2.8	2.62	0.068	0.14
TB	5.9 ± 1.8	7.3 ± 2.0	8.2 ± 3.5	2.95	0.058	0.18
BB	5.2 ± 1.8	4.8 ± 1.9	4.0 ± 2.5	2.28	0.074	0.13
DEL	5.3 ± 1.8	5.6 ± 1.9	5.8 ± 3.0	0.49	0.618	0.03
BRD	4.5 ± 1.6	5.0 ± 1.8	5.3 ± 2.8	1.88	0.167	0.12

Note: Values are presented as mean ± standard deviation. η^2^ = eta squared. *p*-values were adjusted using the Benjamini–Hochberg false discovery rate procedure within the RMS outcome family. b indicates a significant difference between the Weighted Squats and Real Stimulation groups; c indicates a significant difference between the Fake Stimulation and Real Stimulation groups after FDR correction. GLM = gluteus maximus; VM = vastus medialis; VL = vastus lateralis; BF = biceps femoris; GM = medial gastrocnemius; GL = lateral gastrocnemius; ABS = rectus abdominis; LD = latissimus dorsi; TRAP = trapezius; PM = pectoralis major; DEL = deltoid; BB = biceps brachii; TB = triceps brachii; BRD = brachioradialis.

**Table 4:** Comparison of muscle co-activation indices during the Forward Lunge movement cycle among the three groups.

Muscle Pairs	Weighted Squats	Fake Stimulation	Real Stimulation	F-Value	*p*-Value (FDR-Adjusted)	η^2^
VL-BF	0.38 ± 0.07	0.43 ± 0.08	0.45 ± 0.04	2.56	0.171	0.16
GLM-BF	0.22 ± 0.07	0.35 ± 0.08	0.93 ± 0.05 bc	58.42	<0.001	0.78
VM-BF	0.34 ± 0.06	0.34 ± 0.07	0.36 ± 0.10	2.35	0.167	0.14
PM-LD	0.42 ± 0.10	0.44 ± 0.11	0.47 ± 0.13	0.98	0.384	0.05
TB-BB	0.56 ± 0.11	0.59 ± 0.12	0.56 ± 0.03	2.82	0.363	0.18
DEL-LD	0.35 ± 0.10	0.29 ± 0.09	0.36 ± 0.03	2.48	0.271	0.13
ABS-TRAP	0.45 ± 0.10	0.43 ± 0.10	0.42 ± 0.10	0.55	0.581	0.03

Note: Values are presented as mean ± standard deviation. η^2^ = eta squared. *p*-values were adjusted using the Benjamini–Hochberg false discovery rate procedure within the RMS outcome family. b indicates a significant difference between the Weighted Squats and Real Stimulation groups; c indicates a significant difference between the Fake Stimulation and Real Stimulation groups after FDR correction. GLM = gluteus maximus; VM = vastus medialis; VL = vastus lateralis; BF = biceps femoris; GM = medial gastrocnemius; GL = lateral gastrocnemius; ABS = rectus abdominis; LD = latissimus dorsi; TRAP = trapezius; PM = pectoralis major; DEL = deltoid; BB = biceps brachii; TB = triceps brachii; BRD = brachioradialis.

**Table 5:** Number of muscle synergies.

	Weighted Squats	Fake Stimulation	Real Stimulation
Number of collaborations	1.67 ± 0.82	4.83 ± 0.75	5.17 ± 0.41

## Data Availability

The original contributions presented in this study are included in the article. Further inquiries can be directed to the corresponding author.
